# Atopy and Other Sensitivities in Non-Celiac Wheat Sensitivity: Is There an Associated Hypersensitivity Background? †

**DOI:** 10.3390/nu18040609

**Published:** 2026-02-12

**Authors:** Aurelio Seidita, Pasquale Mansueto, Mirco Pistone, Maurizio Soresi, Diana Di Liberto, Marianna Lauricella, Lydia Giannitrapani, Giovanni Pratelli, Giuseppe Mazzarella, Alessandra Camarca, Francesco Maurano, Giuseppe Mogavero, Antonio Carroccio

**Affiliations:** 1 Unit of Internal Medicine, “V. Cervello” Hospital, Ospedali Riuniti “Villa Sofia-Cervello”, Via Trabucco, 180, 90146 Palermo, Italy; aurelio.seidita@unipa.it (A.S.); mirco.pistone@gmail.com (M.P.); 2 Department of Health Promotion Sciences, Maternal and Infant Care, Internal Medicine and Medical Specialties (PROMISE), University of Palermo, 90127 Palermo, Italy; pasquale.mansueto@unipa.it (P.M.); maurizio.soresi@unipa.it (M.S.); lydia.giannitrapani@unipa.it (L.G.); 3 Institute for Biomedical Research and Innovation (IRIB), National Research Council (CNR), 90146 Palermo, Italy; 4 Department of Biomedicine, Neurosciences and Advanced Diagnostics (BIND), Institute of Biochemistry, University of Palermo, 90127 Palermo, Italy; diana.diliberto@unipa.it (D.D.L.); marianna.lauricella@unipa.it (M.L.); giovanni.pratelli@unipa.it (G.P.); 5 Institute of Food Sciences, National Research Council (CNR), 83100 Avellino, Italy; giuseppe.mazzarella@cnr.it (G.M.); alessandra.camarca@cnr.it (A.C.); francesco.maurano@isa.cnr.it (F.M.); 6 Unit of Gastroenterology, “V. Cervello” Hospital, Ospedali Riuniti “Villa Sofia-Cervello”, Via Trabucco, 180, 90146 Palermo, Italy; g.mogavero@villasofia.it

**Keywords:** atopy, food allergies, food sensitivity, non-celiac wheat sensitivity

## Abstract

Background: A hypersensitivity reaction has been hypothesized as one of the possible pathophysiological mechanisms involved in non-celiac wheat sensitivity (NCWS). Some studies have reported a high frequency of atopic diseases in NCWS patients. Objectives: This study aimed (A) to define the presence and features of atopic diseases and other hypersensitivities in NCWS patients and (B) to search for possible allergic features which could address a NCWS diagnosis. Methods: Clinical, laboratory and histological data from NCWS patients before the start of a wheat-free diet were retrospectively analyzed and compared to control subjects with celiac disease (CeD) or irritable bowel syndrome/functional dyspepsia (IBS/FD). Results: Atopic disease prevalence was higher in the NCWS patients (32.8%) than in those with CeD (19.3%) and IBS/FD (21.5%) (*p* = 0.001 for both). Similarly, NCWS subjects reported a higher frequency of multiple food sensitivities (MFSs) (39.8%) and self-reported milk intolerance (SRMI) (65.9%) compared to the control groups (*p* < 0.001 for both). On multiple logistic regression analysis, a coexistent atopic disease (OR 1.481), MFS (OR 3.882) and SRMI (OR 2.259) proved to be variables associated with the NCWS diagnosis. Conclusions: NCWS subjects have a higher frequency of atopic disease, MFS and SRMI when compared to both CeD and IBS/FD patients. All these conditions could be considered as an expression of an underlying hypersensitivity *milieu* characterizing NCWS and might be of support in the differential diagnosis between NCWS and functional gastrointestinal disorders, if inserted into a broader diagnostic panel.

## 1. Introduction

Non-celiac wheat sensitivity (NCWS) is considered a heterogeneous ‘umbrella’ condition [1,2] in which various wheat components might be responsible for intestinal mucosal damage, leading to an increase in intestinal permeability (IP) and exposure of foods/microbial peptides to immune cells, resulting in both a local and systemic inflammatory response [3,4,5,6,7,8]. This hypothetical mechanism could explain both its gastrointestinal (irritable bowel syndrome/functional dyspepsia (IBS/FD)-like) and extraintestinal clinical manifestations, which make this disease similar to other well-known pathologies such as celiac disease (CeD) and IBS/FD [8,9,10,11,12,13,14,15].

CeD is an immune-mediated enteropathy triggered by gluten ingestion in genetically predisposed individuals carrying HLA-DQ2 and/or DQ8 haplotypes, characterized by small intestinal villous atrophy, malabsorption, and specific serological markers [16].

In contrast, IBS and FD are functional gastrointestinal disorders defined by symptom-based criteria (Rome IV), in the absence of structural or biochemical abnormalities, and are not specifically related to wheat or gluten intake. Its physiological basis is still debated, with multiple variables that have been considered as potentially triggering symptoms: visceral hypersensitivity, alteration of intestinal permeability, alterations of the gut microbiota, intake of foods containing Fermentable Oligosaccharides, Disaccharides, Monosaccharides, and Polyols (FODMAPs), and others [17].

The clinical overlap among NCWS, CeD, and IBS/FD—particularly gastrointestinal symptoms such as abdominal pain, bloating, and altered bowel habits—makes differential diagnosis challenging and highlights the need for additional clinical features that may help discriminate among these conditions [18].

So, diagnosing NCWS remains challenging because there is no specific biomarker for it, and diagnosis is based on ruling out CeD and immunoglobulin (Ig) E-mediated wheat allergy (WA), clinical improvement upon applying a wheat-free diet (WFD), and worsening during a double-blind placebo-controlled challenge (DBPCC) with wheat [3,9]. This exclusion-based approach often leads to misdiagnosis and the mislabeling of symptoms, forcing patients on a lengthy and unsatisfactory diagnostic journey. Among NCWS patients, at least two subgroups can be identified: one sensitive only to wheat and one with multiple food sensitivities (MFSs). The latter has characteristics similar to WA but without a positive IgE, thus suggesting a possible non-IgE-mediated immunological response [9]. Previous studies, not specifically designed with this purpose, have indicated a high prevalence of atopic diseases in NCWS patients (29–47%) [9,19,20,21,22,23].

This study therefore aims to (A) better characterize atopic diseases and other hypersensitivity disorders in NCWS patients compared to CeD and IBS/FD ones, as well as to (B) search for possible hypersensitivity features which could address NCWS diagnosis.

## 2. Materials and Methods

This retrospective, multicenter study included NCWS patients consecutively diagnosed by DPBCC with gluten/wheat, between January 2016 and December 2023, in three tertiary centers for the diagnosis and treatment of CeD and food allergies/intolerances: (1) the Internal Medicine Unit of the University Hospital of Palermo, Italy; (2) the Internal Medicine Unit of the ‘Villa-Sofia-Cervello’ Hospital of Palermo, Italy; (3) and the Internal Medicine Unit of the ‘John Paul II Hospital’ of Sciacca, Agrigento, Italy. Patients’ clinical records were retrospectively reviewed together with those of a control population, made up of sex- and age-matched patients suffering from CeD or IBS/FD unrelated to wheat/gluten intake, consecutively diagnosed in the same centers and the same period.

All subjects agreed to the use of their data by signing an informed consent form. This study follows the principles of the Declaration of Helsinki and has been approved by the Ethics Committee of the University Hospital of Palermo (n. 03/2024) and registered on ClinicalTrials.gov (protocol n. NCT06191432, 1 February 2024).

### 2.1. Patient Selection

NCWS was diagnosed according to established criteria, including exclusion of CeD and IgE-mediated WA, clinical response to a WFD (i.e., ≥30% decrease in symptoms compared to baseline), and symptom recurrence/worsening (i.e., ≥30% increase in symptoms compared to WFD) during a DPBCC with wheat [9,24].

CeD diagnosis was based on serology (specifically anti-tissue transglutaminase (tTG) IgA positivity or tTG IgG positivity whenever an IgA deficit was proved), histological findings, and HLA typing according to international guidelines [16,25,26,27].

IBS and FD were diagnosed according to the Rome IV criteria after exclusion of organic gastrointestinal diseases [17].

Patients were included in the study whenever fulfilling the inclusion/exclusion criteria (Supplementary File S1). In particular, all patients without specific allergology tests, or whose atopic disease history was incomplete/unclear were excluded.

As this study is based on real-life clinical practice, it was not possible to perform endoscopy in all the patients included. However, applying the inclusion/exclusion criteria, which required a follow-up of longer than 12 months, we excluded all patients in whom an intestinal and/or extraintestinal disease might have caused the reported symptoms. Moreover, CeD was excluded by the absence of duodenal villous atrophy, documented in all patients carrying the human leukocyte antigen (HLA) DQ2 and/or DQ8 haplotypes, and therefore regardless of the negativity of CeD-specific serum antibodies. To exclude inflammatory bowel diseases, all patients underwent both fecal calprotectin assay and abdominal and intestinal ultrasound scans, and, when clinically required, other imaging and/or endoscopic examinations.

### 2.2. Study Outcomes

To define the prevalence and features of atopic diseases and other hypersensitivities in NCWS patients and compare these data with CeD and IBS/FD controls, the following data were collected:

- Family history of atopic disease: Allergic conjunctivitis, allergic rhinitis, allergic asthma and atopic dermatitis in first/second-degree family members;
- Diagnosis of personal atopic disease: Allergic conjunctivitis, allergic rhinitis, allergic asthma and atopic dermatitis [excluding nickel allergic contact dermatitis (Ni-ACD), which was considered independently] diagnosed according to international criteria [28,29,30,31];
- Identified allergens: House dust mites, *Graminaceae*, wormwood, mugwort, *Salsola kali*, *Parietaria officinalis*, *Olea europea*, *Cupressus*, pollens (unspecified), *Aspergillus*, *Alternaria*, mold (unspecified), and cat/dog/rabbit dander;
- Diagnosis of Ni-ACD: Defined according to the European Society of Contact Dermatitis criteria [32,33];
- Positivity on patch tests by ‘GIRDCA’ (Italian Contact and Environmental Dermatitis Research Group) or ‘SIDAPA’ (Italian Society of Allergological, Occupational and Environmental Dermatology) [34] or the European baseline series [32,33];
- Other food allergies/intolerances:
  ◦ MFSs: Self-reported clinical reactions to foods different from gluten/wheat and milk;
  ◦ Self-reported milk intolerance (SRMI): Self-reported clinical reaction to foods containing milk and milk derivatives (e.g., fresh milk, fresh milk derivatives, etc.);
  ◦ Lactose intolerance: Positive and symptomatic lactose breath test [35];
  ◦ Cow’s milk protein allergy (CMPA): Hypersensitivity reaction involving immunological mechanisms (IgE, non-IgE, and mixed) to cow’s milk proteins and its derivatives [36].
- Skin prick test (SPT) positivity for foods [37];
- Total serum IgE (kU/L) assay;
- Food-specific serum IgE assay positivity [37].

All these variables represent possible clinical and laboratory expressions of a coexisting hypersensitivity reaction in patients enrolled in the study.

It must be underlined that SRMI and MFS were based on self-reported clinical reactions and should therefore be interpreted as subjective clinical features rather than objectively validated diagnoses.

To clarify whether the patient’s ‘atopic signature’ can help in addressing NCWS diagnosis, we analyzed its putative association with the clinical manifestations characterizing NCWS.

### 2.3. Statistical Analysis

Data were expressed as mean ± standard deviation (SD) when the distribution was Gaussian, and Student’s *t*-test was used to evaluate differences between the groups. Otherwise, data were expressed as medians and ranges and analyzed with Mann–Whitney U tests. The χ^2^ test and Fisher’s exact test were used to compare values of frequency in the various population groups.

Multiple logistic regression analysis was performed to estimate (a) the independence of the association between variables significant at univariate analysis and NCWS diagnosis and (b) in NCWS patients, the independence of the association between variables significant at univariate analysis and atopic disease diagnosis. For variables significant in multivariate analysis, the Absolute Risk (AR) was calculated [38].

The SPSS Statistics, version 27.0 (Chicago, IL, USA), and MedCalc, version 22.0 (Acacialaan, Ostend, Belgium), software packages were used for the statistical analysis.

## 3. Results

During the study period, 506 patients were consecutively diagnosed with NCWS, 316 with CeD and 601 with IBS/FD. After the application of inclusion/exclusion criteria, 387 patients with NCWS, 192 patients with CeD, and 303 patients with IBS/FD were enrolled in the study (Supplementary Figure S1). The three groups were comparable in terms of sex distribution and age at diagnosis. Females were prevalent in all groups, particularly among NCWS patients. As expected, gastrointestinal and extraintestinal clinical features differed among groups. IBS-like symptoms were more frequently reported by IBS/FD patients, whereas weight loss and anemia were more prevalent in CeD subjects. Extraintestinal manifestations were more commonly observed in NCWS patients. All CeD patients carried HLA-DQ2 and/or DQ8 haplotypes, while HLA positivity was observed in approximately half of NCWS patients and in one third of IBS/FD subjects. Histological findings reflected the diagnostic criteria of the three diseases, with villous atrophy being observed exclusively in CeD patients. Table A1 (Appendix A) shows patients’ demographic, clinical, and histological features.

### 3.1. Prevalence of Family and Personal Atopy

**Figure 1:**
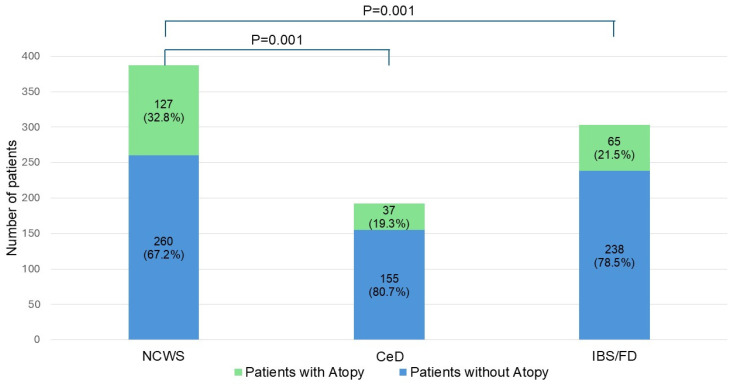
Frequency of atopy in NCWS, CeD and IBS/FD patients. CeD = celiac disease; IBS/FD = irritable bowel syndrome/functional dyspepsia; NCWS = non-celiac wheat sensitivity.

Patients with NCWS (27.9%) and CeD (27.1%) more frequently reported a family history of atopy compared to IBS/FD (8.3%, *p* < 0.001 for both). Among the NCWS patients 32.8% (N = 127) had been diagnosed with an atopic disease, whereas both CeD (19.3%, N = 37) and IBS/FD (21.5%, N = 65) patients reported a significantly lower prevalence of atopy (*p* = 0.001 for both) (Table 1 and Figure 1).

Among the clinical manifestations of atopy, a certain difference was found between NCWS and IBS/FD patients, with the former suffering more often from allergic asthma, either alone (*p* = 0.03) or in association with allergic rhinitis (*p* = 0.005), and the latter from allergic conjunctivitis (*p* = 0.004) or allergic rhinitis alone (*p* = 0.02). No differences were found between the groups concerning the specific type of allergen able to induce symptom onset, except for a higher frequency of *Graminaceae*, which was more evident in patients with NCWS (70.1%) vs. both CeD (48.7%, *p* = 0.03) and IBS/FD (50.8%, *p* = 0.01). Finally, a higher prevalence of Ni-ACD was found in NCWS (17.8%) compared to both CeD (9.4%, *p* = 0.01) and IBS/FD (6.7%, *p* < 0.001), which was also confirmed by the patch tests results (*p* = 0.004 and *p* < 0.001, for NCWS vs. CeD and vs. IBS, respectively; all the subjects with Ni-ACD had patch test positivity to nickel, but the full patch test data also included positivity to other allergens) (Table 1).

### 3.2. Prevalence of Other Food Sensitivities and Intolerances

**Figure 2:**
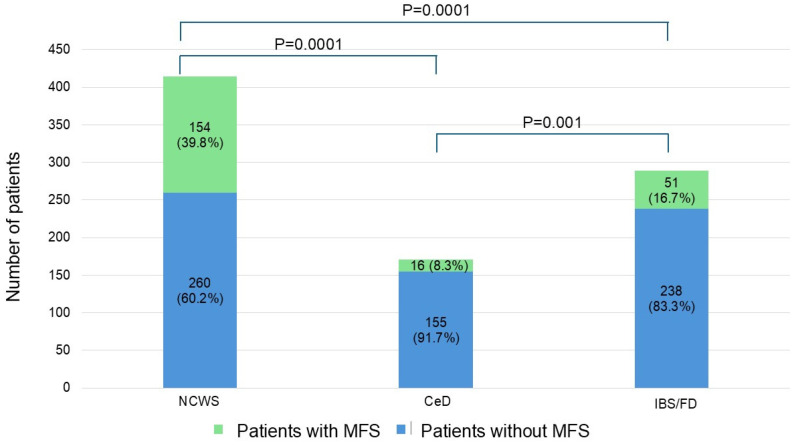
Frequency of MFS in NCWS, CeD and IBS/FD patients. CeD = celiac disease; IBS/FD = irritable bowel syndrome/functional dyspepsia; NCWS = non-celiac wheat sensitivity.

NCWS patients reported a higher frequency of MFS (39.8%) compared to both CeD (8.3%) and to IBS/FD (16.7%) subjects (*p* < 0.001 for both) (Table 1 and Figure 2).

Similarly, compared to the other subgroups, a higher proportion of NCWS subjects complained of SRMI (N = 255, N = 71, and N = 91, respectively, for NCWS, CeD and IBS/FD, *p* < 0.001 for both NCWS vs. CeD and vs. IBS/FD) and were diagnosed with CMPA (N = 19, N = 6, and N = 5, for NCWS, CeD and IBS/FD, respectively, *p* = 0.04 for NCWS vs. IBS/FD), whereas no significant differences were found for lactose intolerance, SPT positivity for foods, total serum IgE or food-specific serum IgE (Supplementary Tables S1 and S2).

### 3.3. Role of Atopy and Other Food Sensitivities/Intolerances in Differential Diagnosis of NCWS

On multiple logistic regression analysis, a diagnosis of coexisting atopic disease (OR 1.481, 95% CI 1.006–2.182, *p* = 0.047), MFS (OR 3.882, 95% CI 2.479–6.077, *p* < 0.001) and SRMI (OR 2.259, 95% CI 1.601–3.189, *p* < 0.001) showed a modest but statistically significant association with NCWS diagnosis, with a small effect size (Table 2).

Moreover, considering the subjectivity of MFS and SRMI, we wanted to verify whether removing these two variables from the analysis could affect the results; a modest but significant association was confirmed (OR 1.633, 95% CI 1.288–2.07, *p* = 0.0001) (Supplementary Table S3). The AR of being affected by an atopic disease, MFS and SRMI in NCWS subjects was calculated as 69%, 69.7% and 61.1%, respectively.

### 3.4. Subgroup Analysis

In a subgroup analysis of the NCWS subjects, patients with atopy compared to those without more frequently showed Ni-ACD (*p* < 0.001) and SRMI (*p* = 0.03), positive patch test (*p* < 0.001), SPT for foods (*p* = 0.001), food-specific serum IgE (*p* = 0.002), and elevated total serum IgE (*p* = 0.02) (Table 3 and Table 4).

On multiple logistic regression analysis, patch test positivity (OR 2.955, 95% CI 1.709–5.109, *p* < 0.001), SPT for foods (OR 5.799, 95% CI 1.823–18.444, *p* = 0.003), and food-specific serum IgE (OR 3.101, 95% CI 1.318–7.298, *p* = 0.001) were confirmed as the main variables differentiating the two subgroups (Table 5).

In this case, too, we wanted to analyze the model by removing the subjective variables (MFS and SRMI), confirming the positivity of both the patch test (OR 1.512, 95% CI 1.157–1.975, *p* = 0.002) and the food-specific serum IgE (OR 1.983, 95% CI 1.433–2.743, *p* = 0.0001), but not SPT positivity (Supplementary Table S4). The AR of testing positive for the patch test, SPT and food-specific serum IgE in NCWS patients with atopic disease diagnosis was 50.6%, 19.1% and 60.7%, respectively.

Supplementary Tables S5–S8 report subgroup analyses between patients with and without a personal history of atopy in both CeD and IBS/FD. Although some differences were found between the subgroups, none of these proved significant on multivariate analysis, except for a higher frequency of extra-intestinal symptoms in IBS patients without a personal history of atopy (OR 0.515, 95% CI 0.314–0.845, *p* = 0.009).

Supplementary Tables S9 and S10 report the comparison between the subgroups of atopic NCWS, CeD and IBS/FD patients. NCWS patients with atopy were more frequently females and complained more often of extraintestinal symptoms, MFS and SRMI. In addition, compared exclusively to IBS/FD patients, they suffered less frequently from dyspepsia and more frequently had HLA DQ2/DQ8 positivity, colonic/rectal eosinophil infiltration, family history of atopy, Ni-ACD diagnosis and patch test positivity.

## 4. Discussion

NCWS is a protean clinical entity in which several physiopathological processes (possibility related to different components of wheat) are activated by a common trigger: intake of wheat flour-based products [1,2,4].

However, due to lack of biomarkers for NCWS diagnosis, patients suffering from this condition do not receive a correct diagnosis and very often consult a great number of different specialists, mainly gastroenterologists and allergologists, without receiving a correct diagnosis.

NCWS subjects often report suffering from MFS, SRMI and atopic diseases [9,22,39]. Conversely, studies conducted on patients with known atopic diseases have shown that eliminating gluten from the diet improves symptoms [39,40,41].

Our research group has repeatedly highlighted this possible association, although in most cases in the context of other research (Supplementary Table S11) [9,20,21,22,23]. The aim of our study was to define the prevalence and specific features of atopic disease and other sensitivities in patients with NCWS in comparison to two control groups of patients representing the two main differential diagnoses to NCWS: CeD and IBS/FD [1,2,8,9]. The secondary aim was to search for the presence of hypersensitivity features which could address a NCWS diagnosis.

It is important to state that the distinctive demographic, clinical, genetic and histological features of our populations (NCWS, CeD and IBS/FD) overlap with those reported in the international literature [1,8,9,12].

The prevalence of atopic diseases was significantly higher in NCWS patients than in those with CeD and IBS/FD. This result substantially confirms our previous reports [9,20,21,22,23], but the current results are strengthened by the study design, which, although retrospective, is aimed at evaluating this aspect specifically.

Even more interesting is the difference in clinical manifestations of atopic diseases between NCWS and IBS/FD patients. The former were diagnosed with forms characterized by bronchial involvement (allergic asthma alone or in association with allergic rhinitis) and the latter with forms more commonly found in the general adult population, affecting the eyes and upper respiratory tract (allergic rhinitis and conjunctivitis). Considering this trend, it could be speculated that the ‘atopic march’ remains ‘frozen’ in its ‘intermediate phase’ in NCWS patients. The ‘atopic march’ is typically characterized by the development of atopic dermatitis and a concomitant sensitization to food and aeroallergens in early childhood, progressing to asthma and allergic conjunctivitis and rhinitis in later childhood. These last two persist in the teenage years and adult life, while asthma disappears [42]. The data from our study indicates that in NCWS patients, or at least in a subgroup of these, a very high hyperreactivity to food-derived antigens persists even in adulthood, with elevated rates of MFS, SRMI and CMPA. We could also hypothesize that in NCWS patients, SRMI is based on a non-IgE-mediated hypersensitivity in all those patients with negative lactose breath test and food-specific serum IgE [43,44]. The high prevalence of patch test positivity and Ni-ACD in NCWS patients may suggest the presence of a hypersensitivity-prone background, potentially involving non-IgE-mediated mechanisms. Nevertheless, these findings cannot be considered direct evidence of food allergy or specific immune pathways and should be interpreted with caution.

Remaining in the realm of speculative hypotheses, all these findings may be interpreted in the context of pathogenetic mechanisms that have been previously hypothesized for NCWS. In fact, if, on the one hand, CeD is characterized by an adaptive immune response to gluten with autoimmune features, and, on the other hand, IBS/FD lacks identifiable immune activation markers, NCWS, considered a protean condition, might occupy an intermediate position. According to some NCWS study groups, there is evidence of innate immune activation and increased intestinal permeability, potentially favoring systemic hypersensitivity responses, which however might not be represented in all subjects and, overall, do not seem sufficient to explain the entire etiopathogenetic mechanism [4,5,6,7,8,44]. However, as no direct markers of intestinal permeability or immune activation were assessed in this study, these considerations should be regarded as speculative. This systemic response would account for both the extraintestinal manifestations of NCWS and the high association with autoimmune diseases [8,9,10,11,12,13,45]. The altered IP, causing a continuous exposure of food/microbial antigens to immune system cells, might explain both, directly, the high prevalence of food-sensitivity and, indirectly, the high prevalence of atopic diseases. These might be sustained by two different possible mechanisms: (1) antigen cross-reactivity [46] and (2) a systemic low-grade inflammatory condition which, in subjects with a personal history of atopic disease, could lead to the persistence or exacerbation of allergic–immunological phenomena developed during childhood. Of note, some studies on animal models have reported that amylase–trypsin inhibitors (ATIs) (known activators of myeloid innate immune cells via TLR-4 interaction) [4] might exacerbate hypersensitivity airway inflammatory responses [4,47], further reinforcing our finding of a high prevalence of asthma in NCWS patients and potentially indicating a link between the gastrointestinal and hypersensitivity manifestations of our patients.

Leaving aside the physiopathological speculations, the finding that emerges seems to be rather clear: subjects with NCWS, or at least a subgroup of them, present more often than ones with CeD and IBS/FD with an immunological background leading to hypersensitivity reactions. This statement is in our opinion supported by the results of the multiple logistic regression analysis. In fact, if the whole population is considered, the features clearly associated with the NCWS diagnosis are atopic disease, MFS and SRMI. Nevertheless, atopy and NCWS are (and must be considered) two distinct clinical entities; our findings suggest that they may share a common hypersensitivity-prone immunological background. While atopy is classically mediated by an IgE-dependent mechanism, NCWS appears to involve both innate and adaptive immune responses, hypothetically mediated by a non-IgE mediated hypersensitivity reaction. The increased prevalence of atopy in NCWS patients may reflect a shared systemic hyperreactivity rather than a direct causal relationship.

Although, it must be clearly stated that even if atopic diseases were significantly more prevalent in NCWS patients, our data does not support the interpretation of NCWS as an allergic disorder. Objective markers of IgE-mediated hypersensitivity, including SPT, food-specific IgE and total serum IgE, were largely comparable among NCWS, CeD and IBS/FD patients. These findings suggest that the observed association reflects a hypersensitivity-prone clinical background (possibly non-IgE mediated) rather than evidence of a classical (igE-mediated) allergic mechanism.

Notwithstanding that NCWS is a heterogeneous disorder including groups of patients with different clinical and immunological features, it can still be stated that our study provides hypothesis-generating evidence that selected clinical features related to hypersensitivity may help characterize subsets of NCWS patients. We suggest that, due to its heterogeneity, it will be difficult to find a single diagnostic biomarker characterizing this condition, and that it will still be necessary to rely on a broad panel to differentiate NCWS patients from those with functional gastrointestinal disorders. In this context, therefore, a subject with gastrointestinal and/or extraintestinal symptoms who self-perceives worsening after an intake of wheat or milk-based foods (i.e., SRMI), for whom an MFS can be found, and who has a diagnosis of atopic disease has a higher probability of having NCWS rather than IBS/FD. Obviously, the associations observed in this study should not be interpreted as diagnostic markers of NCWS. Rather, the presence of atopic disease, MFS and SRMI might be considered a supportive clinical feature that, when integrated into a comprehensive assessment, can increase the pre-test probability of NCWS and assist in differentiating it from functional gastrointestinal disorders. These features are not intended to replace established diagnostic steps, including the exclusion of CeD and IgE-mediated WA, nor the use of dietary intervention and challenge when appropriate. However, in routine clinical practice, their recognition may help clinicians identify patients more likely to benefit from a targeted dietary approach, thereby optimizing diagnostic pathways and avoiding unnecessary or prolonged investigations. Thus, a practical consequence of this association could be to advise patients with the aforementioned features to start a long-term WFD (which may not even be indicated in a patient with a IBS/FD due to the nutritional risks of elimination diets) [48,49] without undergoing the DBPCC with wheat, which is frequently refused by patients.

In fact, atopic disease, MFS and SRMI must not be considered the only components of a new hypothetical NCWS diagnostic panel. Various possible markers have been proposed, from Anti-gliadin IgG antibodies [5] to serum zonulin levels [50] to changes in the gut microbiota [51]. In light of this, it is desirable for individual research groups to join forces, and drawing on experience provided by each group and the diagnostic panels already tested [5,50], design an international and multicenter study that can identify a diagnostic panel including clinical, laboratory and microbiological data which will be unequivocally accepted and simplify NCWS diagnosis.

Overall, our findings do not support the reclassification of NCWS as an allergic (Ig-E-mediated) disease. Rather, they suggest that a subset of NCWS patients exhibits a hypersensitivity-prone clinical phenotype, characterized by an increased prevalence of atopic conditions and self-reported food-related reactions in the absence of consistent IgE-mediated markers.

This study has some limitations: the retrospective design and the characteristics of the recruiting centers introduce an inevitable referral and spectrum bias. The tertiary referral setting of the participating centers represents a potential source of referral and spectrum bias. Patients evaluated in specialized units for wheat-related disorders and food intolerances are more likely to present complex clinical phenotypes and MFS, which may limit the generalizability of our findings to community-based populations. Therefore, these results should be interpreted as hypothesis-generating and warrant confirmation in prospective studies conducted in unselected populations. Another limitation is the impossibility of collecting data which could support the physiopathological explanation behind the findings reported in this study. More specifically, we were not able to obtain data regarding either IP impairment or the immunological activation profile. Finally, it must be acknowledged that MFS and SRMI are subjective variables, based on patient-reported symptoms rather than blinded challenge procedures. As such, they may partially overlap with functional gastrointestinal disorders and symptom perception mechanisms. Thus, it cannot be ruled out that the results of our study are influenced by the subjectivity of some of the parameters evaluated and the setting in which the patients were enrolled. It is therefore necessary to confirm our hypotheses by expanding the sample with a multicenter approach, enrolling an unselected population, and seeking to reduce the impact that ‘subjective’ data have on the analysis.

For all these, our results must be considered preliminary and require further confirmation through prospective studies that can overcome the flaws of the present study and demonstrate the physiopathological bases of our findings.

Nevertheless, the strength of our study is that the NCWS diagnosis was carried out scrupulously using the DBPCC with gluten/wheat and therefore ensuring the certainty of the diagnosis. In addition, this study included a very large cohort of patients enrolled in a tertiary center with recognized experience in NCWS diagnosis; to our knowledge, no studies in this field have investigated such large NCWS, CeD, and IBS/FD populations, exactly reflecting the features described in the international literature.

## 5. Conclusions

In this study, we have specifically proved that NCWS patients have a higher frequency of atopic disease, MFS, and SRMI than both patients with CeD and IBS/FD. All three conditions could be considered as an expression of an underlying hypersensitivity *milieu* which seems to characterize NCWS. These findings indicate that NCWS patients more frequently exhibit atopic conditions and other hypersensitivity traits compared to CeD and IBS/FD. However, the lack of consistent Ig-E-mediated markers suggests that NCWS should not be considered an allergic disorder but rather a condition associated with a hypersensitivity-prone clinical phenotype.

The evidence from this study could be of support in the differential diagnosis between NCWS and functional gastrointestinal disorders, even if it cannot be considered as ‘diagnostic markers’ of the disease.

## Figures and Tables

**Table 1:** Presence of atopy/food allergy and specific related clinical and laboratory characteristics of the patients with NCWS, CeD and IBS/FD enrolled in the study.

	NCWS (N = 387) A	CeD (N = 192) B	IBS/FD (N = 303) C	*p* Value
Family history of atopy, N (%)	108 (27.9)	52 (27.1)	25 (8.3)	A vs. C < 0.001
				B vs. C < 0.001
Allergic conjunctivitis, N (%)	9/108 (8.3)	1/52 (1.9)	4/25 (16.0)	NS
Allergic rhinitis, N (%)	47/108 (43.5)	29/52 (55.8)	5/25 (20.0)	B vs. C 0.007
Allergic asthma, N (%)	27/108 (25.0)	12/52 (23.1)	7/25 (28.0)	NS
Atopic dermatitis, N (%)	4/108 (3.7)	4/52 (7.7)	3/25 (12.0)	NS
Rhinitis + allergic asthma, N (%)	21/108 (19.4)	6/52 (11.5)	6/25 (24.0)	NS
Clinical manifestation of atopy, N (%)	127 (32.8)	37 (19.3)	65 (21.5)	A vs. C 0.001 B vs. C 0.001
Allergic conjunctivitis, N (%)	16 (12.6)	6 (16.2)	20 (30.8)	A vs. C 0.004
Allergic rhinitis, N (%)	46 (36.2)	16 (43.2)	36 (55.4)	A vs. C 0.02
Allergic asthma, N (%)	21 (16.5)	6 (16.2)	3 (4.6)	A vs. C 0.03
Atopic dermatitis, N (%)	11 (8.7)	0(0.0)	1(1.5)	NS
Rhinitis + allergic asthma, N (%)	33 (26.0)	9 (24.3)	5 (7.7)	A vs. C 0.01
Identified allergens	N = 127	N = 37	N = 65	
House dust mites, N (%)	49 (38.6)	16 (43.2)	26 (40.0)	NS
Graminaceae, N (%)	89 (70.1)	18 (48.7)	33 (50.8)	A vs. B 0.03
				A vs. C 0.01
Wormwood, N (%)	6 (4.7)	1 (2.7)	0 (0.0)	NS
Mugwort, N (%)	4 (3.1)	1 (2.7)	3 (4.6)	NS
*Salsola kali*, N (%)	1 (0.8)	0 (0.0)	0 (0.0)	NS
*Parietaria*, N (%)	41 (32.3)	6 (16.2)	19 (29.2)	NS
*Olea europea*, N (%)	21 (16.5)	7 (18.9)	19 (29.2)	NS
*Cupressus*, N (%)	16 (12.6)	4 (10.8)	6 (9.2)	NS
Pollens (unspecified), N (%)	16 (12.6)	3 (8.1)	0 (0.0)	NS
*Aspergillus*, N (%)	1 (0.8)	0 (0.0)	0 (0.0)	NS
*Alternaria*, N (%)	3 (2.4)	0 (0.0)	3 (4.6)	NS
Mold (unspecified), N (%)	2 (1.6)	1 (2.7)	0 (0.0)	NS
Cat dander, N (%)	19 (15.0)	8 (21.6)	8(12.3)	NS
Dog dander, N (%)	10 (7.9)	5 (13.5)	7 (10.8)	NS
Rabbit dander, N (%)	2 (1.6)	0 (0.0)	0 (0.0)	NS
Ni-ACD, N (%)	69 (17.8)	18 (9.4)	20 (6.7)	A vs. B 0.01 A vs. C < 0.001
Patch test				
Negative, N (%)	308 (79.6)	172 (89.6)	283 (93.3)	A vs. B 0.004
Positive, N (%)	79 (20.4)	20 (10.4)	20 (6.7)	A vs. C < 0.001
MFS, N (%)	154 (39.8)	16 (8.3)	51 (16.7)	A vs. B < 0.001 A vs. C < 0.001 B vs. C 0.001
SRMI, N (%)	255 (65.9)	71 (37.0)	91 (30.0)	A vs. B < 0.001 A vs. C < 0.001
Lactose intolerance, N (%)	87/155 (56.1)	30/47 (63.8)	40/80 (50.0)	NS
CMPA, N (%)	19 (4.9)	6 (3.1)	5 (1.7)	A vs. C 0.04
SPT for foods				
Negative, N (%)	366 (94.6)	188 (97.9)	296 (97.7)	NS
Positive, N (%)	21 (5.4)	4 (2.1)	7 (2.3)	
Total serum IgE (kU/L), median (IQR)	83.3 (33.2–303.4)	78.4 (25.5–271.6)	72.4 (25.8–252.3)	NS
Food-specific serum IgE				
Negative, N (%)	359 (92.8)	186 (96.9)	288 (95.0)	NS
Positive, N (%)	28 (7.2)	6 (3.1)	15 (5.0)	

CeD = celiac disease; CMPA = cow’s milk protein allergy; IBS/FD = irritable bowel syndrome/functional dyspepsia; IgE = immunoglobulin E; IQR = interquartile range; MFS = multiple food sensitivities; NCWS = non-celiac wheat sensitivity; Ni-ACD = nickel allergic contact dermatitis; NS = not significant; SPT = skin prick test; SRMI = self-reported milk intolerance.

**Table 2:** Multiple logistic regression: dependent variables, NCWS diagnosis *.

	B	OR	CI 95%	*p*
Diagnosis of atopic disease	0.393	1.481	1.006–2.182	0.047
MFS	1.356	3.882	2.479–6.077	<0.001
SRMI	0.815	2.259	1.601–3.189	<0.001

* NCWS diagnosis compared to non-NCWS diagnosis (i.e., patients diagnosed with either CeD or IBS/FD). CI = confidence interval; CeD = celiac disease; IBS/FD = irritable bowel syndrome/functional dyspepsia; MFS = multiple food sensitivities; NCWS = non-celiac wheat sensitivity; OR = odds ratio; SRMI = self-reported milk intolerance.

**Table 3:** Demographic, clinical and histological characteristics of the NCWS patients enrolled in the study, divided according to the absence or presence of personal history of atopy.

	NCWS Without Atopy (N = 260)	NCWS with Atopy (N = 127)	*p* Value
Gender			
Female, N (%)	228 (87.7)	116 (91.3)	NS
Male, N (%)	32 (12.3)	11 (8.7)	NS
Age at diagnosis (years), mean ± SD	39.1 ± 1.7	36.4 ± 1.2	NS
BMI			
BMI < 18.5, N (%)	18 (6.9)	11 (8.7)	NS
18.5 ≤ BMI ≤ 24.9, N (%)	158 (60.8)	72 (56.7)	NS
25 ≤ BMI ≤ 29.9, N (%)	58 (22.3)	30 (23.6)	NS
BMI ≥ 30, N (%)	26 (10.0)	14 (11.0)	NS
IBS-Like Symptoms			
No symptoms	29 (11.2)	18 (14.2)	NS
Diarrhea, N (%)	116 (44.6)	59 (46.5)	NS
Constipation, N (%)	50 (19.2)	14 (11.0)	NS
Mixed, N (%)	65 (25.0)	36 (28.3)	NS
Dyspepsia, N (%)	150 (57.7)	75 (57.5)	NS
Weight loss, N (%)	78 (30.0)	32 (25.2)	NS
Anemia, N (%)	102 (39.2)	43 (33.9)	NS
Extraintestinal symptoms, N (%)	187 (71.9)	99 (78.0)	NS
Autoimmune diseases, N (%)	78 (30.0)	33 (26.0)	NS
Hashimoto’s thyroiditis, N (%)	47 (18.1)	19 (15.0)	NS
Other autoimmune diseases, N (%)	39 (15.0)	18 (14.2)	NS
Presence of HLA DQ2/DQ8, N (%)	138 (53.1)	63 (49.6)	NS
EGDS, N (%)	196 (75.4)	81 (63.8)	0.02
Marsh Score	N = 196	N = 81	
Marsh 0, N (%)	97 (49.5)	46 (56.8)	NS
Marsh 1, N (%)	95 (48.5)	33 (40.7)	NS
Marsh 2, N (%)	4 (2.0)	2 (2.5)	NS
Duodenal mucosa eosinophils, N (%)	42 (21.4)	11 (13.6)	NS
Colonoscopy, N (%)	105 (40.4)	39 (30.7)	NS
Colonic/rectal mucosa eosinophils, N (%)	27 (25.7)	11 (28.2)	NS

BMI = body mass index; EGD = esophagogastroduodenoscopy; HLA = human leukocyte antigens; IBS = irritable bowel syndrome; NCWS = non-celiac wheat sensitivity; NS = not significant; SD = standard deviation.

**Table 4:** Atopy/hypersensitivity-specific clinical and laboratory characteristics in the NCWS patients enrolled in the study, divided according to the absence or presence of personal history of atopy.

	NCWS Without Atopy (N = 260)	NCWS with Atopy (N = 127)	*p* Value
Family history of atopy, N (%)	64 (24.6)	44 (34.6)	NS
Allergic conjunctivitis, N (%)	4/64 (6.3)	5/44 (11.4)	NS
Allergic rhinitis, N (%)	28/64 (43.8)	19/44 (43.2)	NS
Allergic asthma, N (%)	18/64 (28.1)	9/44 (20.5)	NS
Atopic dermatitis, N (%)	1 /64 (1.6)	3/44 (6.8)	NS
Rhinitis + allergic asthma, N (%)	13/64 (20.3)	8/44 (18.2)	NS
Ni-ACD, N (%)	31 (11.9)	38 (29.9)	<0.001
Patch test			
Negative, N (%)	220 (84.6)	88 (69.3)	<0.001
Positive, N (%)	40 (15.4)	39 (30.7)	
MFS, N (%)	99 (38.1)	55 (43.3)	NS
SRMI, N (%)	161 (61.9)	94 (74.0)	0.03
Lactose intolerance, N (%)	52/93 (55.9)	35/62 (56.5)	NS
CMPA, N (%)	13 (5.0)	6 (4.7)	NS
SPT for foods			
Negative, N (%)	256 (98.5)	110 (86.6)	0.001
Positive, N (%)	4 (1.5)	17 (13.4)	
Total serum IgE (kU/L), median (IQR)	47.9 (10.2–203.3)	228.3 (92.1–351.4)	0.02
Food-specific serum IgE			
Negative, N (%)	249 (95.8)	110 (86.6)	0.002
Positive, N (%)	11 (4.2)	17 (13.4)	

CMPA = cow’s milk proteins allergy; IgE = immunoglobulin E; IQR = interquartile range; MFS = multiple food sensitivities; NCWS = non-celiac wheat sensitivity; Ni-ACD = nickel allergic contact dermatitis; NS = not significant; SPT = skin prick test; SRMI = self-reported milk intolerance.

**Table 5:** Multiple logistic regression: dependent variables, diagnosis of atopic disease in NCWS patients.

	B	OR	CI 95%	*p*
Patch test positivity	1.083	2.955	1.709–5.109	<0.001
SPT for foods positivity	1.758	5.799	1.823–18.444	0.003
Food-specific serum IgE positivity	1.312	3.101	1.318–7.298	0.001

CI = confidence interval; IgE = Immunoglobulin E; NCWS = non-celiac wheat sensitivity; OR = odds ratio; SPT = skin prick test.

## Data Availability

The datasets generated and analyzed during the current study are not publicly available as it belongs to a wider ongoing study funded by the European Union—Next Generation EU, Mission 4, Component 1 CUP B53D23031860001, Project Code: P2022Z9RZY_001, but are available from the corresponding author on reasonable request.

## Supplementary Materials

The following supporting information can be downloaded at https://www.mdpi.com/article/10.3390/nu18040609/s1, File S1: Supplemental Methods; Figure S1: Patient selection; Table S1: Comparison of food allergens identified by SPT for foods in patients with NCWS, CeD and IBS/FD enrolled in the study, listed in alphabetical order; Table S2: Comparison of food allergens identified by food-specific serum IgE assay in patients with NCWS, CeD and IBS/FD enrolled in the study, listed in alphabetical order; Table S3: Multiple logistic regression: dependent variable NCWS diagnosis; Table S4: Multiple logistic regression: dependent variable diagnosis of atopic disease in NCWS patients; Table S5: Demographic, clinical and histological characteristics of CeD patients enrolled in the study divided according to the absence or presence of personal history of atopy; Table S6: Atopy/hypersensitivity-specific clinical and laboratory characteristics of CeD patients enrolled in the study divided according to the absence or presence of personal history of atopy; Table S7: Demographic, clinical and histological characteristics of IBS/FD patients enrolled in the study, divided according to the absence or presence of personal history of atopy; Table S8: Atopy/hypersensitivity-specific clinical and laboratory characteristics of IBS/FD patients enrolled in the study, divided according to the absence or presence of personal history of atopy; Table S9: Comparison of demographic, clinical and histological characteristics of patients with NCWS, CeD and IBS/FD enrolled in the study reporting a personal history of atopy; Table S10: Comparison of atopy-specific clinical and laboratory characteristics of patients with NCWS, CeD and IBS/FD enrolled in the study reporting a personal history of atopy; Table S11: Prevalence of atopic diseases in patients with NCWS, CeD and IBS/FD reported by previous studies of our group.

## Abbreviations

The following abbreviations are used in this manuscript:

ATIs	amylase–trypsin inhibitors
CeD	celiac disease
CMPA	cow’s milk protein allergy
DBPCC	double-blind placebo-controlled challenge
FD	functional dyspepsia
FODMAPs	Fermentable Oligosaccharides, Disaccharides, Monosaccharides, and Polyols
GFD	gluten-free diet
HLA	human leukocyte antigens
IBS	irritable bowel syndrome
IgE	immunoglobulin E
IgG	immunoglobulin G
IP	intestinal permeability
IQR	interquartile range
MFS	multiple food sensitivities
NCWS	non-celiac wheat sensitivity
Ni-ACD	nickel allergic contact dermatitis
SD	standard deviation
SPT	skin prick test
SRMI	self-reported milk intolerance
TLR-4	toll-like receptor 4
WA	IgE-mediated wheat allergy
WFD	wheat-free diet

## Appendix A

**Table A1:** Demographic, clinical, genetic and histological characteristics of the patients with NCWS, CeD and IBS/FD enrolled in the study.

	NCWS (N = 387) A	CeD (N = 192) B	IBS/FD (N = 303) C	*p* Value
Gender				
Female, N (%)	344 (88.9)	152 (79.2)	253 (83.5)	NS
Male, N (%)	43 (11.1)	40 (20.8)	50 (16.5)	NS
Age at diagnosis (years) (mean ± SD)	37.9 ± 1.9	38.1 ± 15.3	40.8 ± 14.0	NS
IBS-like symptoms, N (%)	340 (87.9)	144 (75.0)	298 (98.3)	A vs. B < 0.001 A vs. C < 0.001 B vs. C < 0.001
Dyspepsia, N (%)	223 (57.6)	109 (56.8)	177 (58.3)	NS
Weight loss, N (%)	110 (28.4)	78 (40.6)	81 (21.7)	A vs. B 0.05 B vs. C 0.002
Anemia, N (%)	145 (37.5)	106 (52.2)	66 (26.7)	A vs. B < 0.001 A vs. C < 0.001 B vs. C < 0.001
Extraintestinal symptoms, N (%)	286 (73.9)	118 (61.5)	152 (50.0)	A vs. B 0.03 A vs. C < 0.001 B vs. C 0.02
Autoimmune diseases, N (%)	111 (28.7)	42 (21.9)	51 (16.7)	A vs. C < 0.001
Presence of HLA DQ2/DQ8, N (%)	201 (51.9)	192 (100)	96 (31.7)	A vs. B < 0.001 A vs. C < 0.001 B vs. C < 0.001
EGDS, N (%)	277 (71.6)	192 (100.0)	86 (28.3)	A vs. B < 0.001 B vs. C < 0.001 A vs. C < 0.001
Marsh Score	N = 277	N = 192	N = 86	
Marsh 0, N (%)	143 (51.6)	0 (0.0)	76 (88.4)	A vs. B < 0.001 A vs. C < 0.001 B vs. C < 0.001
Marsh 1, N (%)	134 (48.4)	0 (0.0)	10 (11.6)	A vs. B < 0.001 A vs. C < 0.001 B vs. C < 0.001
Marsh 2, N (%)	0 (0.0)	0 (0.0)	0 (0.0)	N/A
Marsh 3A, N (%)	0 (0.0)	40 (20.8)	0 (0.0)	A vs. B < 0.001 B vs. C < 0.001
Marsh 3B, N (%)	0 (0.0)	58 (30.2)	0 (0.0)	A vs. B < 0.001 B vs. C < 0.001
Marsh 3C, N (%)	0 (0.0)	94 (49.0)	0 (0.0)	A vs. B < 0.001 B vs. C < 0.001
Duodenal mucosa eosinophils, N (%)	N = 277 53 (19.1)	N = 192 24 (12.5)	N = 86 10 (11.6)	NS
Colonoscopy, N (%)	144 (37.2)	30 (15.6)	96 (31.7)	A vs. B < 0.001 B vs. C < 0.001
Colonic/rectal mucosa eosinophils, N (%)	N = 144 38 (26.4)	N = 30 7 (23.3)	N = 96 5 (5.2)	A vs. C < 0.001 B vs. C 0.009
