# Supplementary material for: Atopy and Other Sensitivities in Non-Celiac Wheat Sensitivity: Is There an Associated Hypersensitivity Background?
Source: Nutrients. 2026 Feb 12;18(4):609. doi: 10.3390/nu18040609 (PMC12943107; doi:10.3390/nu18040609)
Supplement: Supplementary file 1 [file nutrients-18-00609-s001.zip › Supplementary Tables 10_02_2026.pdf]

Supplementary Table 1. Comparison of food allergens identified by SPT for foods in the patients with NCWS, CeD and IBS/FD enrolled in the study, listed in alphabetical order.

	**NCWS (N=21) A**	**CeD (N=4) B**	**IBS/FD (N=7) C**	***P value***
Almonds	2 (9.5)	0 (0.0)	1 (14.3)	NS
Celery	2 (9.5)	0 (0.0)	0 (0.0)	NS
Cocoa	1 (4.8)	0 (0.0)	0 (0.0)	NS
Cod	1 (4.8)	0 (0.0)	0 (0.0)	NS
Corn	3 (14.3)	1 (25.0)	0 (0.0)	NS
Egg	4 (19.5)	0 (0.0)	2 (28.6)	NS
Fish	3 (14.3)	1 (25.0)	0 (0.0)	NS
Garlic	1 (4.8)	0 (0.0)	0 (0.0)	NS
Hazelnuts	2 (9.5)	2 (50.0)	1 (14.3)	NS
Legumes	0 (0.0)	1 (25.0)	0 (0.0)	NS
Lettuce	1 (4.8)	0 (0.0)	0 (0.0)	NS
LTP	1 (4.8)	0 (0.0)	0 (0.0)	NS
Milk	0 (0.0)	1 (25.0)	0 (0.0)	NS
Onion	2 (9.5)	1 (25.0)	1 (14.3)	NS
Peas	0 (0.0)	1 (25.0)	0 (0.0)	NS
Peanuts	2 (9.5)	0 (0.0)	2 (28.6)	NS
Potato	1 (4.8)	0 (0.0)	0 (0.0)	NS
Sardines	0 (0.0)	1 (25.0)	0 (0.0)	NS
Shrimp	1 (4.8)	0 (0.0)	1 (14.3)	NS
Stone fruits	0 (0.0)	1 (25.0)	0 (0.0)	NS
Strawberries	1 (4.8)	0 (0.0)	0 (0.0)	NS
Tomato	3 (14.3)	1 (25.0)	0 (0.0)	NS
Walnuts	2 (9.5)	0 (0.0)	1 (14.3)	NS
Yeast	1 (4.8)	0 (0.0)	0 (0.0)	NS

CeD=celiac disease; IBS/FD=irritable bowel syndrome/functional dyspepsia; NCWS=non-celiac wheat sensitivity; NS=not significant; LTP=lipid transfer protein; SPT=skin prick test.

Supplementary Table 2. Comparison of food allergens, identified by food-specific serum IgE assay in the patients with NCWS, CeD and IBS/FD enrolled in the study, listed in alphabetical order.

	NCWS (N=28) A	CeD (N=6) B	IBS/FD (N=15) C	*P value*
Almonds	2 (7.1)	0 (0.0)	1 (6.7)	NS
Apple	1 (3.6)	0 (0.0)	1 (6.7)	NS
Apricots	2 (7.1)	0 (0.0)	1 (6.7)	NS
Banana	3 (10.7)	1 (16.7)	0 (0.0)	NS
Carrots	0 (0.0)	1 (16.7)	0 (0.0)	NS
Cherries	1 (3.6)	0 (0.0)	0 (0.0)	NS
Egg	9 (32.1)	2 (33.3)	0 (0.0)	NS
Fennel	1 (3.6)	0 (0.0)	0 (0.0)	NS
Fish	5 (17.9)	1 (16.7)	1 (6.7)	NS
Garlic	3 (10.7)	0 (0.0)	3 (20.0)	NS
Hazelnuts	4 (14.3)	0 (0.0)	2 (13.3)	NS
Latex	2 (7.1)	0 (0.0)	1 (6.7)	NS
Lemons	1 (3.6)	0 (0.0)	0 (0.0)	NS
Lentils	1 (3.6)	1 (16.7)	0 (0.0)	NS
Maize	1 (3.6)	0 (0.0)	0 (0.0)	NS
Milk	0 (0.0)	4 (66.7)	3 (20.0)	NS
Onion	1 (3.6)	0 (0.0)	1 (0.0)	NS
Oranges	1 (3.6)	0 (0.0)	0 (0.0)	NS
Peas	1 (3.6)	0 (0.0)	1 (6.7)	NS
Peanuts	2 (7.1)	1 (16.7)	1 (6.7)	NS
Potato	1 (3.6)	0 (0.0)	0 (0.0)	NS
Shrimp	2 (7.1)	1 (16.7)	1 (6.7)	NS
Soy	2 (7.1)	0 (0.0)	1 (6.7)	NS
Spinach	1 (3.6)	0 (0.0)	1 (6.7)	NS
Strawberries	2 (7.1)	0 (0.0)	2 (13.3)	NS
Tomato	5 (17.9)	1 (16.7)	1 (6.7)	NS
Walnuts	5 (17.9)	0 (0.0)	2 (13.3)	NS
Yeast	1 (3.6)	1 (0.0)	3 (20.0)	NS

CeD=celiac disease; IBS/FD=irritable bowel syndrome functional dyspepsia; IgE=immunoglobulin E; NCWS=non-celiac wheat sensitivity; NS=not significant.

Supplementary Table 3. Multiple logistic regression: dependent variable NCWS diagnosis*.

	**B**	**OR**	**CI 95%**	***P***
Diagnosis of atopic disease	0.49	1.633	1.288-2.07	0.0001

**NCWS diagnosis compared to non-NCWS diagnosis (i.e. patients diagnosed as suffering from either CeD or IBS/FD). The following variables were excluded from analysis due to their self-reported nature: MFS and SRMI.*

CI=confidence interval; CeD=celiac disease; IBS/FD=irritable bowel syndrome/functional dyspepsia; MFS=multiple food sensitivities; NCWS=non-celiac wheat sensitivity; OR=odds ratio; SRMI=self-reported milk intolerance.

Supplementary Table 4. Multiple logistic regression: dependent variable diagnosis of atopic disease in NCWS patients.*.

	**B**	**OR**	**CI 95%**	***P***
Patch test	0.413	1.512	1.157-1.975	0.002
Food-specific serum IgE positivity	0.684	1.983	1.433-2.743	0.0001

** The following variables were excluded from analysis due to their self-reported nature: MFS and SRMI.*

CI=confidence interval; Ig=immunoglobulin; MFS=multiple food sensitivities; NCWS=non-celiac wheat sensitivity; OR=odds ratio; SRMI=self-reported milk intolerance.

Supplementary Table 5. Demographic, clinical and histological characteristics of the CeD patients enrolled in the study divided according to the absence or presence of personal history of atopy.

	**CeD without atopy (N=155)**	**CeD with atopy (N=37)**	***P value***
Gender			
Female, N (%)	124 (80.0)	28 (75.7)	NS
Male, N (%)	31 (20.0)	9 (24.3)	NS
Age at diagnosis (years), mean ± SD	39.3 ± 15.5	33.0 ± 13.4	NS
IBS-like symptoms, N (%)	115 (74.2)	29 (78.4)	NS
Dyspepsia, N (%)	91 (58.7)	18 (48.6)	NS
Weight loss, N (%)	61 (39.4)	17 (45.9)	NS
Anemia, N (%)	87 (56.1)	19 (51.4)	NS
Extraintestinal symptoms, N (%)	96 (61.9)	22 (59.5)	NS
Autoimmune diseases, N (%)	35 (22.6)	7 (18.9)	NS
Presence of HLA DQ2/DQ8, N (%)	155 (100.0)	37 (100.0)	NS
EGDS, N (%)	155 (100.0)	37 (100.0)	NS
Marsh Score			
Marsh 3A, N (%)	34 (21.9)	6 (16.2)	NS
Marsh 3B, N (%)	47 (30.3)	11 (29.7)	NS
Marsh 3C, N (%)	74 (47.7)	20 (54.1)	NS
Duodenal mucosa eosinophils, N (%)	20 (12.9)	4 (10.8)	NS
Colonoscopy, N (%)	24 (15.5)	6 (16.2)	NS
Colonic/rectal mucosa eosinophils, N (%)	6 (25.0)	1 (16.7)	NS

BMI=body mass Index; CeD=celiac disease; EGDS=esophagogastroduodenoscopy; HLA=human leukocyte antigens; IBS=irritable bowel syndrome; IQR=interquartile range; NS=not significant.

Supplementary Table 6. Atopy/hypersensitivity-specific clinical and laboratory characteristics of the CeD patients enrolled in the study divided according to the absence or presence of personal history of atopy.

	**CeD without atopy (N=155)**	**CeD with atopy (N=37)**	***P value***
Family history of atopy, N (%)	36 (23.2)	16 (43.2)	0.03
Allergic conjunctivitis, N (%)	1/36 (2.8)	0/16 (0.0)	NS
Allergic rhinitis, N (%)	21/36 (58.3)	8/16 (50.0)	NS
Allergic asthma, N (%)	7/36 (19.4)	5/16 (31.3)	NS
Atopic dermatitis, N (%)	3/36 (8.3)	1/16 (6.3)	NS
Rhinitis + Allergic asthma, N (%)	4/36 (11.1)	2/16 (12.5)	NS
Ni-ACD, N (%)	10 (6.5)	8 (21.6)	0.01
Patch test			
Negative, N (%)	143 (92.3)	29 (78.4)	0.03
Positive, N (%)	12 (7.7)	8 (21.6)	
MFS, N (%)	9 (5.8)	7 (18.9)	0.02
SRMI, N (%)	54 (34.8)	17 (14.9)	NS
Lactose intolerance, N (%)	23/31 (74.2)	7/16 (43.7)	NS
CMPA, N (%)	4 (2.6)	2 (5.4)	NS
SPT for foods			
Negative, N (%)	154 (99.3)	34 (91.9)	0.03
Positive, N (%)	1 (0.7)	3 (8.1)	
Total serum IgE (kU/L), median (IQR)	37.1 (11.4-161.3)	189.6 (89.9-334.2)	0.02
Food-specific serum IgE			
Negative, N (%)	151 (97.4)	35 (94.6)	NS
Positive, N (%)	4 (2.6)	2 (5.4)	

CeD=celiac disease; CMPA=cow's milk protein allergy; IgE=immunoglobulin E; IQR=interquartile range; MFS=multiple food sensitivities; Ni-ACD=nickel allergic contact dermatitis; NS=not significant; SPT=skin prick test; SRMI=self-reported milk intolerance.

Supplementary Table 7. Demographic, clinical and histological characteristics of the IBS/FD patients enrolled in the study, divided according to the absence or presence of personal history of atopy.

	**IBS/FD without atopy (N=238)**	**IBS/FD with atopy (N=65)**	***P value***
Gender<br>Female, N (%)<br>Male, N (%)	 205 (86.1)<br>33 (13.9)	 48 (73.8)<br>17 (26.2)	 0.03
Age at diagnosis (years), mean ± SD	42.1 ± 15.0	38.1 ± 11.5	NS
IBS-like symptoms, N (%)	234 (98.3)	64 (98.5)	NS
Dyspepsia, N (%)	128 (53.8)	49 (75.4)	0.003
Weight loss, N (%)	50 (21.0)	16 (24.6)	NS
Anemia, N (%)	31 (13.0)	14 (21.5)	NS
Extraintestinal symptoms, N (%)	133 (55.9)	19 (29.2)	<0.001
Autoimmune diseases, N (%)	41 (17.2)	10 (15.4)	NS
Presence of HLA DQ2/DQ8, N (%)	81 (34.0)	15 (23.1)	NS
EGDS, N (%)	76 (31.9)	10 (15.4)	<0.01
Marsh score<br>Marsh 0, N (%)<br>Marsh 1, N (%)	N=76<br>69 (90.8)<br>7 (9.2)	N=10<br>7 (70.0)<br>3 (30.0)	 NS
Duodenal mucosa eosinophils, N (%)	N=76<br>9 (11.8)	N=10<br>1 (10.0)	 NS
Colonoscopy, N (%)	80 (33.6)	16 (24.6)	NS
Colonic/rectal mucosa eosinophils, N (%)	N=71<br>4 (5.6)	N=16<br>1 (6.3)	 NS

BMI=body mass index; EGDS=esophagogastroduodenoscopy; HLA=human leukocyte antigens; IBS/FD=irritable bowel syndrome/functional dyspepsia; NS=not significant; SD=standard deviation.

Supplementary Table 8. Atopy/hypersensitivity-specific clinical and laboratory characteristics of the IBS/FD patients enrolled in the study, divided according to the absence or presence of personal history of atopy.

	**IBS/FD without atopy (N=238)**	**IBS/FD with atopy (N=65)**	***P value***
Family history of atopy, N (%)	15 (6.3)	10 (15.4)	0.04
Allergic conjunctivitis, N (%)	2/15 (13.3)	2/10 (20.0)	NS
Allergic rhinitis, N (%)	3/15 (20.0)	2/10 (20.0)	NS
Allergic asthma, N (%)	4/15 (26.6)	3/10 (30.0)	NS
Atopic dermatitis, N (%)	2/15 (13.3)	1/10 (10.0)	NS
Rhinitis + Allergic asthma, N (%)	4/15 (26.6)	2/10 (20.0)	NS
Ni-ACD, N (%)	14 (5.9)	6 (9.2)	NS
Patch test			
Negative, N (%)	224 (94.1)	59 (90.8)	NS
Positive, N (%)	14 (5.9)	6 (9.2)	
MFS, N (%)	35 (14.7)	16 (24.6)	NS
SRMI, N (%)	68 (28.6)	23 (35.4)	NS
Lactose intolerance, N (%)	28/59 (47.5)	12/21 (57.1)	NS
CMPA, N (%)	2 (0.8)	3 (4.6)	NS
SPT for foods			
Negative, N (%)	234 (98.3)	62 (95.4)	NS
Positive, N (%)	4 (1.7)	3 (4.6)	
Total serum IgE (kU/L), median (IQR)	44.1 (10.3- 159.2)	198.2 (85.2-322.4)	0.02
Food-specific serum IgE			
Negative, N (%)	229 (96.2)	59 (90.8)	NS
Positive, N (%)	9 (3.8)	6 (9.2)	

CMPA=cow's milk protein allergy; IBS/FD=irritable bowel syndrome/functional dyspepsia; IgE=immunoglobulin E; IQR=interquartile range; MFS=multiple food sensitivities; Ni-ACD=nickel allergic contact dermatitis; NS=not significant; SPT=skin prick test; SRMI=self-reported milk intolerance.

Supplementary Table 9. Comparison of demographic, clinical and histological characteristics of the patients with NCWS, CeD and IBS/FD enrolled in the study reporting a personal history of atopy.

	**NCWS with atopy (N=127) A**	**CeD with atopy (N=37) B**	**IBS/FD with atopy (N=65) C**	***P value***
Gender				
Female, N (%)	116 (91.3)	28 (75.7)	48 (73.8)	A *vs* B 0.02
Male, N (%)	11 (8.7)	9 (24.3)	17 (26.2)	A vs C 0.002
Age at diagnosis (years) (mean ± SD)	36.4±1.2	33.0±13.4	38.1±11.5	NS
IBS-like symptoms, N (%)	109 (85.8)	29 (78.4)	64 (98.5)	A *vs* C 0.01<br>B *vs* C 0.002
Dyspepsia, N (%)	75 (57.5)	18 (48.6)	49 (75.4)	A *vs* C 0.04<br>B *vs* C 0.01
Weight loss, N (%)	32 (25.2)	17 (45.9)	16 (24.6)	A *vs* B 0.03<br>B *vs* C 0.04
Anemia, N (%)	43 (33.9)	19 (51.4)	14 (21.5)	B *vs* C 0.04
Extraintestinal symptoms, N (%)	99 (78.0)	22 (59.5)	19 (29.2)	A *vs* B 0.04<br>A *vs* C <0.001<br>B *vs* C 0.005
Autoimmune diseases, N (%)	33 (26.0)	7 (18.9)	10 (15.4)	NS
Presence of HLA DQ2/DQ8, N (%)	63 (49.6)	37 (100.0)	15 (23.1)	A *vs* B <0.001<br>A *vs* C <0.001<br>B *vs* C <0.001
EGDS, N (%)	81 (63.8)	37 (100.0)	10 (15.4)	A *vs* B <0.001<br>A *vs* C <0.001<br>B *vs* C <0.001
Marsh Score	N=81	N=37	N=10	
Marsh 0, N (%)	46 (56.8)	0 (0.0)	7 (70.0)	A *vs* B <0.001
Marsh 1, N (%)	35 (43.2)	0 (0.0)	3 (30.0)	A *vs* B <0.001
Marsh 2, N (%)	0 (0.0)	0 (0.0)	0 (0.0)	N/A
Marsh 3A, N (%)	0 (0.0)	6 (16.2)	0 (0.0)	A *vs* B <0.001<br>B *vs* C <0.001
Marsh 3B, N (%)	0 (0.0)	11 (29.7)	0 (0.0)	A *vs* B <0.001<br>B *vs* C <0.001
Marsh 3C, N (%)	0 (0.0)	20 (54.1)	0 (0.0)	A *vs* B <0.001<br>B *vs* C <0.001
Duodenal mucosa eosinophils, N (%)	N=81<br>11 (13.6)	N=37<br>4 (10.8)	N=10<br>1 (10.0)	NS
Colonoscopy, N (%)	39 (30.7)	6 (16.2)	16 (24.6)	NS
Colonic/rectal mucosa eosinophils, N (%)	N=39<br>11 (28.2)	N=6<br>1 (16.7)	N=16<br>1 (6.3)	A *vs* C 0.04

BMI=body mass index; CeD=celiac disease; EGDS=esophagogastroduodenoscopy; HLA=human leukocyte antigens; IBS/FD=irritable bowel syndrome/functional dyspepsia; NCWS=non-celiac wheat sensitivity; N/A=not applicable; NS=not significant; SD=standard deviation.

Supplementary Table 10. Comparison of atopy-specific clinical and laboratory characteristics of the patients with NCWS, CeD and IBS/FD enrolled in the study reporting a personal history of atopy.

	**NCWS with atopy (N=127) A**	**CeD with atopy (N=37) B**	**IBS/FD with atopy (N=65) C**	***P value***
Family history of atopy, N (%)	44 (34.6)	16 (43.2)	10 (15.4)	A *vs* C 0.008<br>B *vs* C 0.004
Allergic conjunctivitis, N (%)	5/44 (11.4)	0/16 (0.0)	2/10 (20.0)	NS
Allergic rhinitis, N (%)	19/44 (43.2)	8/16 (50.0)	2/10 (20.0)	NS
Allergic asthma, N (%)	9/44 (20.5)	5/16 (31.3)	3/10 (30.0)	NS
Atopic dermatitis, N (%)	3/44 (6.8)	1/16 (6.3)	1/10 (10.0)	NS
Rhinitis + Allergic asthma, N (%)	8/44 (18.2)	2/16 (12.5)	2/10 (20.0)	NS
Ni-ACD, N (%)	38 (29.9)	8 (21.6)	6 (9.2)	A *vs* C 0.002
Patch test				
Negative, N (%)	88 (69.3)	29 (78.4)	59 (90.8)	A *vs* C 0.002
Positive, N (%)	39 (30.7)	8 (21.6)	6 (9.2)	
MFS, N (%)	55 (43.3)	7 (18.9)	16 (24.6)	A *vs* B 0.001<br>A *vs* C 0.02
SRMI, N (%)	94 (74.0)	17 (14.9)	23 (35.4)	A *vs* B 0.002<br>A *vs* C <0.001
Lactose intolerance, N (%)	35/62 (56.5)	7/16 (43.7)	12/21 (57.1)	NS
CMPA, N (%)	6 (4.7)	2 (5.4)	3 (4.6)	NS
SPT for foods				
Negative, N (%)	110 (86.6)	34 (91.9)	62 (95.4)	NS
Positive, N (%)	17 (13.4)	3 (8.1)	3 (4.6)	
Total serum IgE (kU/L), median (IQR)	228.3 (92.1-351.4)	189.6 (89.9-334.2)	198.2 (85.2-322.4)	NS
Food-specific serum IgE				
Negative, N (%)	110 (86.6)	35 (94.6)	59 (90.8)	NS
Positive, N (%)	17 (13.4)	2 (5.4)	6 (9.2)	NS

CeD=celiac disease; CMPA=cow's milk protein allergy; IBS/FD=irritable bowel syndrome/functional dyspepsia; IgE=immunoglobulin E; IQR=interquartile range; MFS=multiple food sensitivities; NCWS=non-celiac wheat sensitivity; Ni-ACD=nickel allergic contact dermatitis; NS=not significant; SPT=skin prick test; SRMI=self-reported milk intolerance.

Supplementary Table 11. Prevalence of atopic diseases in patients with NCWS, CeD and IBS/FD reported by previous studies of our group.

Study, year and study design	Patients with NCWS	Patients with CeD	Patients with IBS/FD	*P value*
Carroccio et al. 2012 (1), Retrospective	29%	8%	6%	NCWS *vs* CeD <0.001<br>NCWS *vs* IBS <0.001<br>CeD *vs* IBS NS
Carroccio et al. 2014 (2), Prospective	30%	20%	9.5%	NCWS *vs* CeD NS<br>NCWS *vs* IBS 0.01<br>CeD *vs* IBS NS
Carroccio et al. 2015 (3),<br>Retrospective	30%	10%	6%	NCWS *vs* CeD 0.001<br>NCWS *vs* IBS 0.001<br>CeD *vs* IBS NS
Prospective	33%	15%	8%	NCWS *vs* CeD NS<br>NCWS *vs* IBS 0.01<br>CeD *vs* IBS NS
D'Alcamo et al. 2017 (4), Prospective	42%	-	8.7%	NCWS *vs* IBS <0.001
Mansueto et al. 2019 (5), Retrospective	40%	39%	-	NCWS *vs* CeD NS

CeD=celiac disease; IBS/FD=irritable bowel syndrome/functional dyspepsia; N/A=not applicable; NCWS=non-celiac wheat sensitivity; NS=not significant

References

1. Carroccio, A.; Mansueto, P.; Iacono, G.; Soresi, M.; D'Alcamo, A.; Cavataio, F.; Brusca, I.; Florena, A.M.; Ambrosiano, G.; Seidita, A. Non-celiac wheat sensitivity diagnosed by double-blind placebo-controlled challenge: Exploring a new clinical entity. *Am. J. Gastroenterol.* **2012**, 107, 1898–1906.
2. Carroccio, A.; Soresi, M.; D'Alcamo, A.; Cavataio, F.; Brusca, I.; Florena, A.M.; Ambrosiano, G.; Seidita, A.; Iacono, G.; Mansueto, P. Risk of low bone mineral density and low body mass index in patients with non-celiac wheat sensitivity: A prospective observation study. *BMC Med.* **2014**, 12, 230.
3. Carroccio, A.; D'Alcamo, A.; Cavataio, F.; Soresi, M.; Seidita, A.; Sciumè, C.; Geraci, G.; Iacono, G.; Mansueto, P. High proportions of people with nonceliac wheat sensitivity have autoimmune disease or antinuclear antibodies. *Gastroenterology* **2015**, 149, 596–603.e1.
4. D'Alcamo, A.; Mansueto, P.; Soresi, M.; Iacobucci, R.; Geraci, G.; Fayer, F.; Seidita, A.; Carroccio, A. Contact dermatitis due to nickel allergy in patients suffering from non-celiac wheat sensitivity. *Nutrients* **2017**, 9, 103.
5. Mansueto, P.; Soresi, M.; La Blasca, F.; Fayer, F.; D'Alcamo, A.; Carroccio, A. Body mass index and associated clinical variables in patients with non-celiac wheat sensitivity. *Nutrients* **2019**, 11, 1220.
