# Supplementary material for: Atopy and Other Sensitivities in Non-Celiac Wheat Sensitivity: Is There an Associated Hypersensitivity Background?
Source: Nutrients. 2026 Feb 12;18(4):609. doi: 10.3390/nu18040609 (PMC12943107; doi:10.3390/nu18040609)
Supplement: Supplementary file 1 [file nutrients-18-00609-s001.zip › Supplementary Figure 1 04_02_2026.pdf]

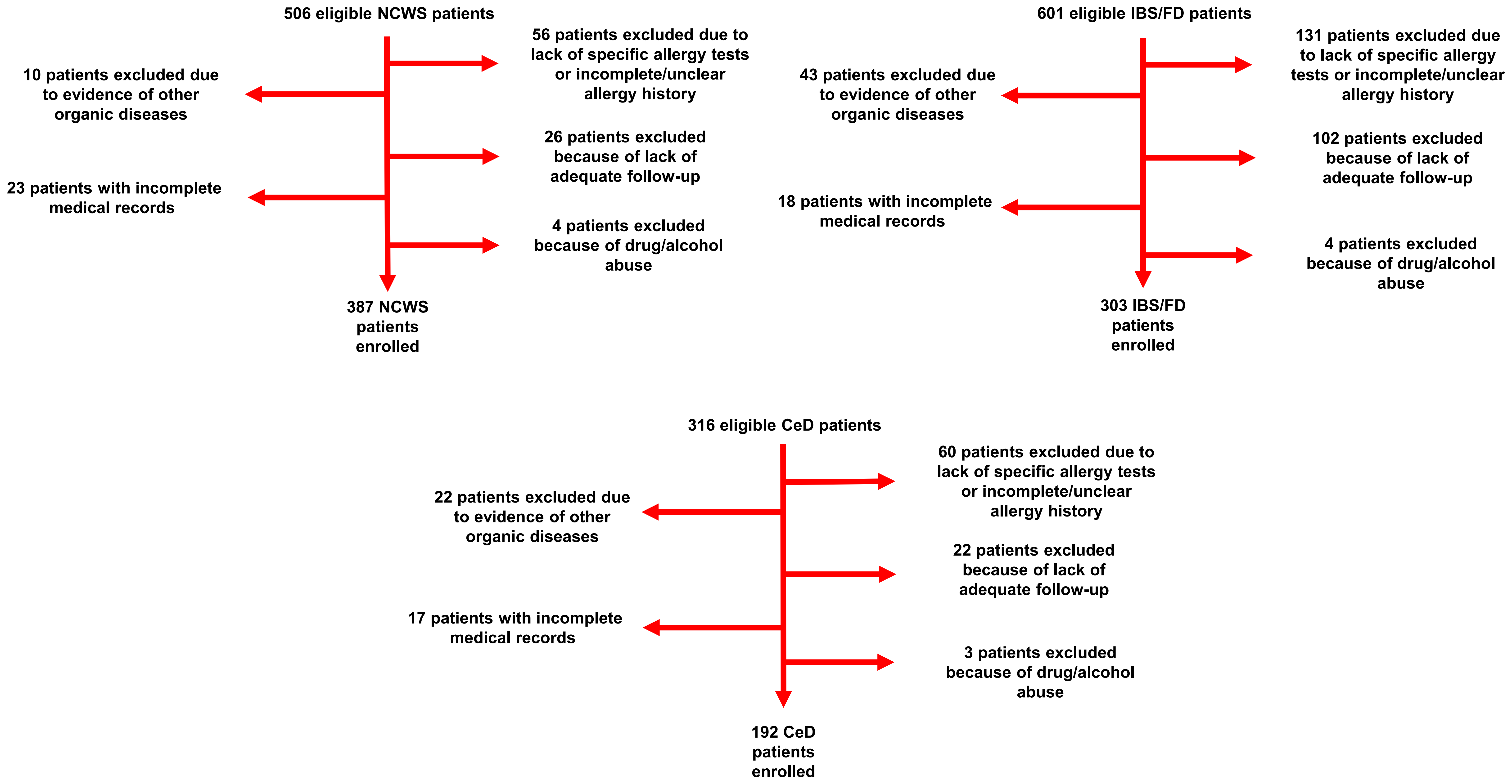

Supplementary Figure 1: Patients selection
CeD: celiac disease; IBS/FD: irritable bowel syndrome/functional dyspepsia; NCWS: non-celiac wheat sensitivity
