# Supplementary material for: Atopy and Other Sensitivities in Non-Celiac Wheat Sensitivity: Is There an Associated Hypersensitivity Background?
Source: Nutrients. 2026 Feb 12;18(4):609. doi: 10.3390/nu18040609 (PMC12943107; doi:10.3390/nu18040609)
Supplement: Supplementary file 1 [file nutrients-18-00609-s001.zip › Supplementary File 1 04_02_2026.pdf]

Supplementary Methods

Diagnostic criteria

Non-celiac wheat sensitivity (NCWS) patients

- subjects with wheat-dependent symptoms, both gastrointestinal and extraintestinal;
- negativity of anti-deamidated gliadin peptide (DGP) immunoglobulin (Ig)A and IgG antibodies, anti-tissue transglutaminase (tTG) IgA and IgG antibodies, and anti-endomysial antibodies (EMA);
- absence of duodenal villous atrophy in all patients carrying the human leukocyte antigen (HLA) DQ2 and/or DQ8 haplotypes (therefore regardless of the negativity of CeD-specific serum antibodies), evaluated when the patients had consumed a minimum of 100g of pasta and/or bread a day, for at least 45 days;
- absence of wheat allergy (WA): negative skin prick test and/or specific serum immunoglobulin E (IgE) assay for wheat, gluten and gliadin;
- resolution of symptoms on a strict standard elimination diet (i.e. extended oligoantigenic, excluding wheat, cow's milk, egg, tomato and chocolate and other foods self-reported by the patient as causing symptoms), followed for at least 4 weeks, and the recurrence of the same symptoms after a double-blind placebo-controlled challenge (DBPCC) with wheat (for further details see below).

Patients who had the following features during the diagnostic workup were not considered suitable for NCWS diagnosis:

- self-exclusion of wheat from the diet and refuse to reintroduce it for diagnostic purposes;
- drug and/or alcohol (>30 g/day for men and >20 g/day for women) abuse;
- treatment with steroids and/or non-steroidal anti-inflammatory drugs in the 2 weeks before duodenal biopsy;
- pregnancy or breastfeeding;
- diagnosis of chronic inflammatory bowel disease or other organic pathologies affecting the digestive system (e.g., WA, microscopic colitis, diverticulitis, segmental colitis associated with diverticulosis,

etc.), neurological diseases, major psychiatric disorders, infectious diseases, immunological deficiencies, and impairments limiting physical activity.

<u>Celiac Disease (CeD) patients</u>

- subjects with gastrointestinal and extraintestinal wheat-dependent symptoms that meet the diagnostic criteria of CeD [1]: positivity of anti-tTG IgA and/or IgG antibodies and evidence of villous atrophy, according to the Marsh-Oberhuber classification, demonstrated by histology on duodenal biopsy [2,3];
- clinical response to a gluten-free diet (GFD) (i.e., resolution of gastrointestinal and/or extraintestinal symptoms).

<u>Irritable bowel syndrome/functional dyspepsia (IBS/FD) and other functional gastrointestinal disorders unrelated to wheat/gluten intake patients</u>

- subjects diagnosed with IBS/FD and other functional gastrointestinal disorders, according to the Rome IV classification [4], who did not specifically report gastrointestinal or extraintestinal symptoms/signs following ingestion of wheat and who did not respond to a wheat-free diet (WFD).

Standard elimination diet and double-blind placebo-controlled challenge (DBPCC)

To diagnose NCWS, all patients in whom other pathologies had already been excluded (e.g., CeD), were asked to follow a standard elimination diet, i.e., an extended oligoantigenic diet, excluding wheat, cow's milk, yeast, eggs, tomato, and chocolate. Patients self-reporting multiple food sensitivities (MFS) were also asked to avoid ingestion and/or contact with other food(s) causing symptoms. Food diaries were kept during the elimination diet period to assess dietary intake and adherence to the diet. After 4 weeks of elimination diet, food challenges were performed, with the reintroduction of a single food at a time. Patients were randomized to receive either the 'active food' or the placebo, according to a computer-generated order determined by an observer not involved in the study.

The DBPCC with wheat was performed with sachets of flour coded A or B containing wheat flour or rice flour, respectively. Sachets A or B were given for 2 consecutive weeks, and then, after 1 week of washout, patients received the other sachets for another 2 weeks (cross-over design). If necessary, the washout period was extended for a maximum of a further 2 weeks until the symptoms induced by the previous challenge had completely resolved before the next challenge was started. Wheat challenges were performed by administering a daily dose of 80g of flour, which was dissolved and cooked by the patients themselves. Wheat sachets contained 6.5g of gluten, and an estimated 0.3g of amylase trypsin inhibitors (ATIs), as determined by bioassay.

The codes of the sachets were broken only at the end of the study and the investigators did not know their contents during the study period. In patients with suspected MFS, challenges for other foods were performed in an open fashion.

During the challenge period, the severity of the intestinal symptoms was recorded: patients completed a 100mm visual analog scale (VAS, with 0 representing no symptoms, and 10 intolerable symptoms), which assessed overall symptoms and the specific symptoms they each reported. The challenges were stopped when clinical reactions occurred for at least two consecutive days (increase in VAS score >30, both for IBS-like symptoms - onset of abdominal discomfort or pain, associated with a change in stool frequency and/or in stool appearance - and for extraintestinal symptoms). Challenges were considered positive if the symptoms which had been initially present reappeared after their disappearance on the elimination diet, and if the VAS score was >30 when compared to any eventual increase determined during the placebo administration.

Inclusion criteria

- age >18 and <65 years;
- specific allergy tests performed during diagnostic work-up (i.e. patch test, total IgE, skin prick tests for foods, food specific IgE)

Exclusion criteria

- incomplete medical records;
- incomplete/unclear personal allergy history;
- lack of clinical follow-up for at least 12 months after diagnosis with >2 outpatient visits during the follow-up period;
- diagnosis of organic diseases such as chronic inflammatory bowel disease or other organic pathologies affecting the digestive system (e.g., WA, microscopic colitis, diverticulitis, segmental colitis associated with diverticulosis, etc.), neurological diseases, major psychiatric disorders, infectious diseases, immunological deficiencies, during the follow-up period.
- drug/alcohol abuse started or discovered during the follow-up period.

Demographic, clinical, genetic and histological characteristics analyzed for the secondary outcome

The following variables were recorded and analyzed: sex, age at diagnosis (years), body mass index [BMI, Kg/m^2, classified according to World Health Organization (WHO) classes], presence and type of IBS presentation (diarrhea, constipation, mixed bowel movements), presence of dyspepsia, weight loss (defined as a 10% reduction in body weight in the last six months or less), anemia (defined as a reduction in hemoglobin levels to less than 13.5 g/dL in men, less than 12.0 g/dL in women), other extraintestinal symptoms and autoimmune diseases (with indication of the specific associated disease), presence of HLA DQ2/DQ8 haplotypes, duodenal histology (classified according to Marsh-Oberhuber) [2,3], and presence of eosinophils in the duodenal, colonic and rectal mucosa.

References

1. Caio, G.; Volta, U.; Sapone, A.; Leffler, D.A.; De Giorgio, R.; Catassi, C.; Fasano, A. Celiac disease: A comprehensive current review. *BMC Med.* **2019**, 17, 142.
2. Marsh, M.N. Gluten, major histocompatibility complex, and the small intestine: A molecular and immunobiologic approach to the spectrum of gluten sensitivity (celiac sprue). *Gastroenterology* **1992**, 102, 330–354.
3. Oberhuber, G.; Granditsch, G.; Vogelsang, H. The histopathology of coeliac disease. *Eur. J. Gastroenterol. Hepatol.* **1999**, 11, 1185–1194.
4. Drossman, D.A.; Hasler, W.L. Rome IV—Functional GI disorders: Disorders of gut–brain interaction. *Gastroenterology* **2016**, 150, 1257–1261.
